# Post-Bariatric Hypoglycemia: Diagnosis, Mechanisms and Management—A Case Report-Based Review

**DOI:** 10.3390/jcm15093220

**Published:** 2026-04-23

**Authors:** Rui Ribeiro, Carina Rossoni, Cláudia Rocha, Octávio Viveiros, Viorel Taranu, Filipa Eiró, Raquel Sousa, Paulo Reis Esselin de Melo, Victor Ramos Mussa Dib, Carlos Augusto Scussel Madalosso, Luciana El Kadre

**Affiliations:** 1 Multidisciplinary Centre for the Treatment of Obesity and Diabetes, Hospital Lusíadas Amadora, 2724-002 Amadora, Portugal; ruijsribeiro@gmail.com (R.R.); octavioviveiros@gmail.com (O.V.); viorel.taranu70@gmail.com (V.T.); filipa_eiro@yahoo.com (F.E.); raquelfdsousa@gmail.com (R.S.); 2 Multidisciplinary Centre for the Treatment of Obesity and Diabetes, Hospital Lusíadas Lisboa, 1500-458 Lisboa, Portugal; 3 Institute of Environmental Health (ISAMB), Faculty of Medicine, University of Lisbon, 1649-026 Lisbon, Portugal; 4 Réseau Hospitalier Neuchâtelois—RHNe, 2000 Neuchâtel, Switzerland; claudia.lopes@hin.ch; 5 APDP—Portuguese Association for the Protection of Diabetics, 1250-203 Lisbon, Portugal; 6 Paulo Reis Institute of Bariatric Surgery, Goiânia 74120-020, Brazil; paulo.3522@gmail.com; 7 Alfredo Nasser University Center (UNIFAN), Goiânia 74905-020, Brazil; 8 Victor Dib Institute, Manaus 69020-210, Brazil; victormussadib@gmail.com; 9 Gastrobese Clinic, Passo Fundo 99010-111, Brazil; gbbariatrica@gmail.com; 10 Brazilian Society of Video Surgery, Robotics and Digital (SOBRACIL), Rio de Janeiro 22631-004, Brazil; lelkadre@gmail.com

**Keywords:** hypoglycemia, Roux-en-Y Gastric Bypass, SADI-S, ileal surgery, review

## Abstract

**Background:** Post-bariatric hypoglycemia (PBH) is a clinically significant complication of bariatric surgery, characterized by inappropriate postprandial hyperinsulinemia and recurrent hypoglycemia. Episodes are often frequent, severe, and medically refractory, substantially impacting quality of life and potentially causing compensatory carbohydrate intake that leads to weight regain. **Methods:** A 50-year-old male underwent Roux-en-Y gastric bypass (RYGB) in 2009. Symptomatic postprandial hypoglycemia emerged in the second postoperative year and progressively worsened to multiple severe daily episodes. The patient developed compensatory carbohydrate intake with subsequent weight regain. Following the failure of dietary interventions and pharmacologic therapy, he underwent conversion to single-anastomosis duodeno-ileostomy with sleeve gastrectomy (SADI-S) in September 2022. **Results:** Following surgical conversion, the patient reported no clinically significant hypoglycemia during the follow-up period. Weight and obesity-related comorbidities improved. Gastrointestinal symptoms remained manageable, and micronutrient status was closely monitored. **Conclusions:** In selected patients with severe, medically refractory PBH following RYGB, conversion to an ileal-based procedure may be considered a viable therapeutic strategy. Prospective studies are needed to better define this hypothesis.

## 1. Introduction

Roux-en-Y gastric bypass (RYGB) is among the most commonly performed bariatric operations worldwide for the treatment of severe obesity [1]. Beyond its robust effects on weight loss and the remission of obesity-related comorbidities, RYGB may be associated with late complications. One of the most clinically relevant complications is post-bariatric hypoglycemia (PBH), characterized by inappropriate postprandial hyperinsulinemia and recurrent hypoglycemia [2,3]. Low plasma glucose levels are clinically important because they may cause cognitive impairment and can progress to loss of consciousness, seizures, or coma [4].

PBH is most frequently recognized after RYGB [5,6,7], but it has also been described after other bariatric and metabolic procedures, including sleeve gastrectomy (SG), one-anastomosis gastric bypass, and duodenal switch [2,8,9,10]. While there are various theories regarding the etiology of the PBH phenomenon, under the most common interpretation it is a late-onset metabolic complication caused by excessive postprandial insulin secretion following surgical alterations to nutrient supply and entero-insular signaling.

No single test provides a definitive PBH diagnosis, and many patients may have asymptomatic episodes [11,12]. PBH is diagnosed when typical postprandial neuroglycopenic and/or adrenergic symptoms occur in association with biochemically confirmed hypoglycemia (plasma glucose < 54 mg/dL) and resolve after glycemia normalization (Whipple’s triad) [13]. Traditionally, a mixed-meal tolerance test (MMTT) is preferred over an oral glucose tolerance test (OGTT) for provocation testing, as it more accurately reflects postprandial physiology in real-world conditions and carries a lower risk of causing severe hypoglycemia. However, more recent guidelines now advise against using OGTT and MMTT for diagnostic purposes [10,11]

Although continuous glucose monitoring (CGM) is widely used in practice, the same guidelines do not recommend it as a primary diagnostic test due to reduced accuracy in the hypoglycemic range and a lack of standardized diagnostic thresholds. However, it may be useful for monitoring and management. When suspicions of PBH episodes are raised, a confirmatory blood glucose test is required. It is also useful to evaluate the duration of episodes and related conditional effects, such as meal composition, exercise intensity, or sleep.

Management is often complex and variably effective. Nutritional therapy remains the cornerstone of treatment. When dietary strategies fail and pharmacologic and/or endoscopic approaches prove ineffective, revisional surgery may be considered in selected, severe, medically refractory cases [10].

In this text, we discuss current trends in PBH diagnosis and the pathophysiological concepts behind management, based on a clinical case exhibiting behavioral and pharmacological intractability that was resolved through the surgical conversion of an RYGB to a SADI-S. A literature review was performed, and the results of this were also included in the report.

The review aimed to identify the clinical characteristics, diagnostics, and therapeutic recommendations for PBH. Articles, guidelines, other reviews, and pathophysiological research studies were targeted. Databases such as PubMed, Medline, and ResearchGate were searched from June to November 2025. The search terms were: ‘hypoglycaemia’; ‘hyperinsulinaemia’; ‘Roux-en-Y gastric bypass’; ‘SADI-S’; ‘bile acids’; ‘ileal surgery’; and ‘metabolic surgery’. The results of this review are presented in this text.

## 2. Case Report

In June 2022, a 50-year-old male (weight 129 kg, height 1.72 m, body mass index [BMI] 43.6 kg/m^2^) was referred to our diabetology clinic. He had undergone RYGB 13 years earlier (pre-operative BMI 45.3 kg/m^2^). Baseline comorbidities included obstructive sleep apnea (OSA), hypertension, dyslipidemia, fibromyalgia, and depression. A weight nadir of 72 kg was reached 2 years postoperatively.

Post-bariatric hypoglycemia (PBH) began 5 years after RYGB. The diagnosis was established based on Whipple’s triad during an OGTT at another hospital. Dietary modification and acarbose provided a partial, transient benefit. Over the two years preceding referral, episodes worsened to multiple (>7) severe daily events with recurrent syncope and significant weight regain (reaching 130 kg), driven by frequent carbohydrate intake to manage symptoms. An oral glucose tolerance test (OGTT) with concomitant insulin measurement (75 g glucose load) was performed following an eight-hour fast. The results confirmed postprandial hyperinsulinemia, with insulin values of 683 µIU/mL, 306 µIU/mL, and 120 µIU/mL at 60, 90, and 120 min, respectively. This was accompanied by hypoglycemia, with a blood glucose reading of 50 mg/dL at 120 min. These results confirmed the diagnosis of PBH.

Additional endocrine investigations were performed at our unit (Table 1 and Table 2).

Trials of verapamil and nifedipine were ineffective. Acarbose provided a slight but transient improvement. Liraglutide, semaglutide, and empagliflozin were attempted in different clinical settings but were discontinued due to intolerance and/or persistent symptoms. The patient was therefore categorized as medically refractory PBH, with complete weight regain plus major functional impairment and referred to our unit for further evaluation and treatment.

Preoperative assessment confirmed severe, disabling hypoglycemia fulfilling Whipple’s triad, a BMI of 44 kg/m^2^, and micronutrient deficiencies, which were corrected prior to surgery. Psychological and anesthesiology evaluations revealed no contraindications (American Society of Anesthesiologists [ASA] physical status II). Importantly, perioperative management included specialized nutritional consultations and the implementation of the Enhanced Recovery After Surgery—Bariatric Surgery (ERAS-BS) protocol [14]. The aim was to improve glycemic stability and optimize nutritional status prior to surgery.

**Figure 1:**
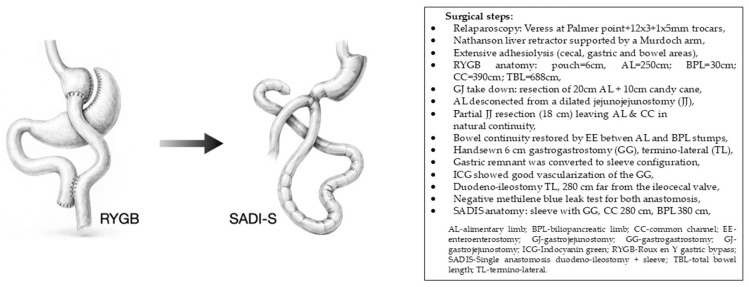
Conversion of a RYGB to a SADI-S.

Given the poor and unpredictable outcomes associated with the most common therapeutic options for PBH, we opted for a conversion to SADI-S, based on the rationale outlined in the following sections. The surgical configuration and intraoperative technical details are described in Figure 1.

**Figure 2:**
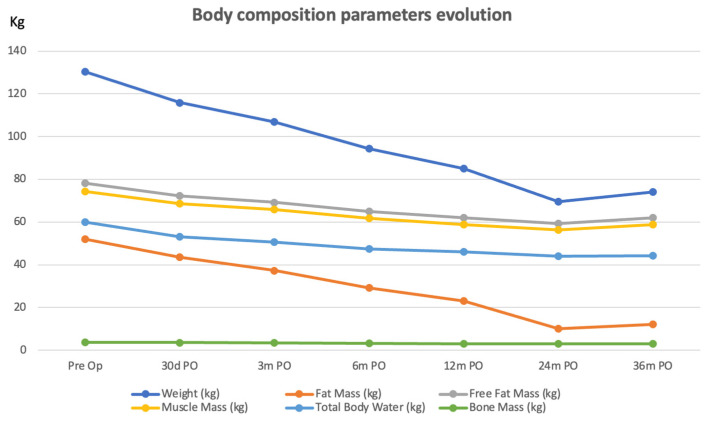
Weight and parameters of body composition evolution over 3 years.

The operative course and recovery were uneventful. A multidisciplinary follow-up protocol was implemented. Weight decreased from 130.3 kg to 74.1 kg (nadir: 69.6 kg) at three years post-procedure. Fat mass, evaluated via bioelectrical impedance analysis (BIA; Tanita DC-430MA S^®^, Tanita Corp., Tokyo, Japan), decreased markedly with relative preservation of lean mass (Figure 2). Bone mass decreased from 3.8 kg to 3.0 kg at 24 months, with a subsequent increase to 3.1 kg at 36 months.

**Figure 3:**
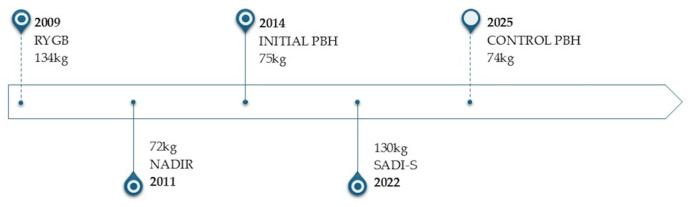
Patient condition timeline.

Glycemic control improved substantially. At 22 months postoperatively, only two episodes of mild symptomatic hypoglycemia occurred, both following unusually prolonged physical exertion. No other symptoms suggestive of PBH occurred during a follow-up period of >3 years (Figure 3). Insulin and C-peptide levels decreased significantly, with corresponding improvements in HOMA-IR and HOMA-β.

Nutritional status was monitored as part of routine postoperative surveillance, and deficiencies were addressed through structured supplementation (Table 2 and Table 3). Deficiencies were mitigated through specialized supplementation tailored for biliopancreatic diversions, including WLS Maximum^®^, Soft Chew Calcium^®^ and vitamin A^®^.

Despite good adherence, hematological assessment revealed a reduction in hematocrit from the first postoperative year onwards, accompanied by a slight but non-significant decrease in hemoglobin.

It is worth noting that these types of deficits are common following SADI-S and other ileal-based procedures. Although bowel adaptation and serum level recovery may occur years later, further research and improved care are needed to address these shortcomings. In our patient, these conditions have not yet led to any significant clinical complaints. Hypertension resolved within three months, and Obstructive Sleep Apnea Syndrome—OSAS within six months. No other physiological or anatomical complications were observed.

## 3. Discussion

### 3.1. Epidemiology and Clinical Relevance

The prevalence of PBH after RYGB remains uncertain, with wide variability across studies, largely reflecting differences in detection methods and diagnostic criteria. Lee et al. (2015) reported that 34% of patients described symptoms consistent with PBH [15]. In contrast, studies using provocation testing (OGTT/MMTT) or CGM have reported rates ranging from 10% to 80% [11,16,17]. Conversely, self-reported data limited to cases requiring hospital admission for recurrent episodes suggest a much lower frequency (≈0.1%) [8,10,18,19,20]. Regardless of prevalence estimates, severe PBH is clinically consequential, with the potential for neuroglycopenic events and substantial quality-of-life impairment.

Therapeutic management is frequently challenging and often only partially effective. This case highlights the medical intractability of PBH, which results in a severe deterioration in quality of life and requires surgical intervention.

### 3.2. Diagnostic Considerations and Differential Diagnosis

PBH is primarily a postprandial phenomenon. Consequently, routine investigation aimed at excluding insulinoma is generally not indicated as a standard step in typical postprandial PBH presentations [10]. Even if not framed as a standard requirement, in this case, abdominal CT and abdominal MRI were performed and did not reveal suspicious duodenal or pancreatic lesions or other relevant abnormalities.

### 3.3. Pathophysiology Considerations

**Figure 4:**
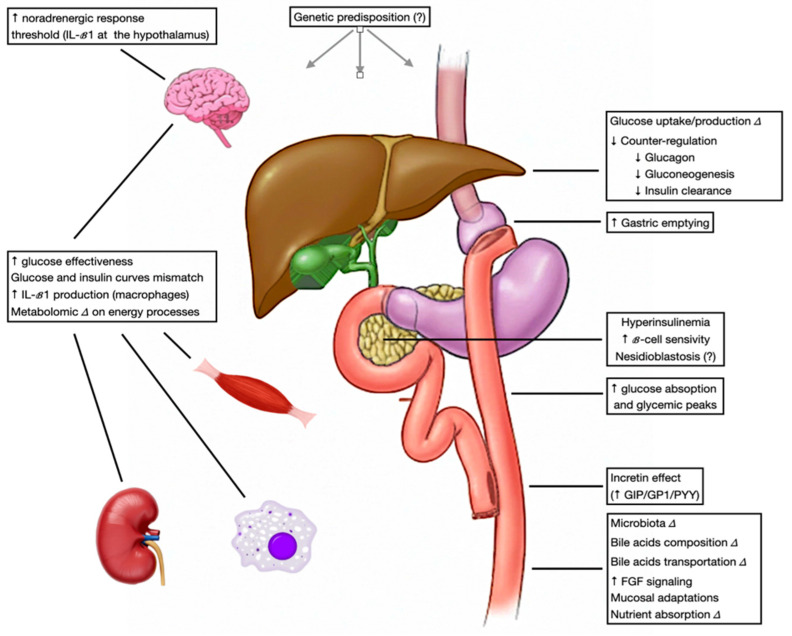
PBH potential mechanisms.

The precise pathophysiology of PBH is not fully established. Over time, multiple, non-exclusive hypotheses have been proposed, including exaggerated postprandial glucose and insulin excursions [5], excessive β-cell responses [12], impaired counter-regulatory hormonal and neural responses [21], changes in gut microbiota [22], bile-acid dysmetabolism [23], bowel mucosal adaptations [24] and, in some reports, nesidioblastosis [12]. Overall, PBH is best considered a complex condition arising from alterations at multiple levels of the postprandial cascade within the gut–liver–pancreas axis (Figure 4).

From a clinical perspective, recognition of risk factors associated with PBH after gastric bypass is important. Reported factors include female sex; younger age, absence of preoperative T2D; absence of preoperative hypoglycemic symptoms; lower BMI, greater postoperative weight loss and prior cholecystectomy. These variables may warrant consideration when selecting procedures and counseling patients [10,15,20,25,26,27].

A useful conceptual framework is to define PBH as a disorder of inappropriate postprandial insulin exposure, insulin secretion and/or persistence that is excessive relative to prevailing glycemic requirements. To accomplish this, several mechanisms have been proposed, and they are not mutually exclusive [12,28,29]: accelerated postprandial glucose absorption leading to a steep early glycemic excursion with a disproportionate insulin response; β-cell hyperresponsiveness with impaired suppression after the peak; delayed suppression and/or reduced clearance of insulin prolonging insulin action beyond glucose availability; impaired counter-regulation (including insufficient glucagon and/or autonomic responses) permitting insulin dominance in the late postprandial window; and abnormalities in hepatic glucose handling in which hepatic glucose output and uptake do not compensate appropriately while insulin remains elevated.

A commonly invoked RYGB-specific explanation emphasizes rapid delivery of carbohydrate-rich nutrients into the alimentary limb, with fast absorption and early glucose spikes. This is followed by an exaggerated incretin-associated insulin response and suppression of glucagon and/or inadequate counter-regulation, potentially culminating in a late postprandial glucose nadir [24]. This model is consistent with the clinical observation that PBH is typically postprandial and can be prominent after bypass procedures.

Pharmacologic observations have also been discussed in this context. Some reports suggest that glucagon-like peptide 1 receptor agonists (GLP-1 Ras), such as liraglutide or semaglutide, may reduce glycemic variability in PBH by modifying postprandial dynamics [30]. Mechanistic explanations proposed in the literature include altered synchronization between glucose absorption and islet hormone responses and a decrease in excessive incretin-driven insulin secretion [31]. However, clinical responses are heterogeneous, and these observations do not establish a single dominant mechanism.

Important limitations of a purely incretin-centered model have been raised. First, PBH has been reported after SG at frequencies described as comparable to other procedures in some studies [9], despite generally lower incretin responses than RYGB [32]. Second, reversal of RYGB to normal anatomy does not consistently resolve PBH [8,12], suggesting that additional mechanisms may contribute materially in at least a subset of patients.

### 3.4. Bile Acids, Fibroblast Growth Factor 19 (FGF 19), and Emerging Hypotheses

The role of bile acids (BA) signaling in PBH remains incompletely defined, but it has received increasing attention. BA absorption may occur within the Biliopancreatic limb (BPL) through the expression of organic anion-transporting polypeptides (OATPs), even in the absence of fat micelles [33,34].

Clinical data have described an association between BPL length and circulating BA levels [35,36] with implications for metabolic outcomes. PBH is described as a late phenomenon and has been reported more frequently in individuals with greater postoperative weight loss [12]. Several time-dependent adaptations have been proposed, including continuous ileal mucosal stimulation and hypertrophy [9] and progressive changes in ileal signaling with increases in GLP-1 and FGF 19 over 1–2 years [25,37]. In cases with much later onset, as in the current patient, later timing may be related to behavioral changes and progressive weight loss rather than a single early mucosal adaptation window.

Studies evaluating BA and FGF 19 after RYGB have reported that total postprandial BA levels may not differ between individuals with and without post-RYGB hypoglycemia, whereas FGF19 has been reported as higher in PBH cases despite similar BA concentrations. More recently, Chaudhari et al. (2025) described a pattern in which PBH cases exhibited higher postprandial plasma BA levels and lower fecal BA content than individuals without PBH, along with higher circulating FGF 19 [29]. They also reported differences in fecal and plasma BA profiles between PBH and non-PBH groups. In this model, BA profiles are proposed to more robustly stimulate FGF 19 production, with stimulation dependent on apical sodium-dependent bile acid transporter (ASBT) function.

In animal model studies, cited in this context, ASBT inhibition with elobixibat reduced FGF 15 expression (the rodent analogue of human FGF 19) and increased postprandial glycemia; whether this relates to altered intestinal glucose absorption due to intraluminal BA changes or to reduced suppression of hepatic glucose production via FGF 19 requires clarification [38,39]. These observations support BA–FGF 19 signaling as a plausible contributor to PBH pathophysiology in some patients, while also underscoring that causal pathways and clinical translation remain to be determined.

Additional observations include reports that enhancing postprandial BA secretion via vagal activation selectively increased prandial unconjugated bile acids in hypoglycemic cases [40]. Collectively, these findings identify BA signaling as an active area of investigation and a potential avenue for therapeutic exploration, without yet establishing a definitive mechanistic hierarchy.

### 3.5. Revisional Surgery and the Rationale for Ileal-Based Strategies

In ileal-based procedures such as SADI-S and BPD/DS, nutrient delivery dynamics differ from RYGB, and PBH has been reported less commonly. Sessa et al. (2019) [5] evaluated postprandial responses after SADI-S and BPD and reported lower glucose absorption and insulin responses to MMT compared with RYGB and SG [5]. In their series of nine patients, none presented with PBH. While limited by small sample size, these data are consistent with the hypothesis that some ileal-based configurations may reduce the postprandial insulin–glucose mismatch implicated in PBH.

In mechanistic terms, the intended physiologic effect of revisional ileal procedures in severe PBH is to reduce the postprandial insulin–glucose mismatch by altering nutrient delivery and intestinal signaling, while also modifying enterohepatic signaling pathways that may influence postprandial metabolic regulation, including BA–FGF 19 signaling. With SADI-S [5], duodenal bipartition [41] or ileal interposition [42], the food delivery process preserves pyloric pacing effect and benefits of the ileal limited sugar absorption capacity.

Given the general limited long-term data for revisional approaches and reports of recurrence after PBH, but based on the previous considerations, an ileal-based revision was preferred in our case. Among available ileal surgical options, conversion from RYGB to SADI-S was selected.

### 3.6. Clinical Considerations

From a clinical perspective, PBH is primarily managed with structured dietary and behavioral measures, which are sufficient for many patients. When nutritional intervention is inadequate, pharmacologic therapy may be considered.

The nutritional approach involves dietary and behavioural changes- These changes resolve the majority of cases. Initially, consists of: (1) identifying and treating nutritional deficiencies; (2) the intake of controlled portions of carbohydrates (CH); (3) eating small, frequent meals, (4) reducing the CH content, choosing low-glycaemic index carbohydrates, only in the context of consuming a single food source; (5) considering a reduction in carbohydrate targets under supervision; (6) the use of corn starch supplements or food additives to modify the GI should be avoided; (7) caffeine consumption requires attention; (8) alcohol intake is discouraged [10].

It should be noted that blood glucose monitoring must be continuous and combined with food recording, in order to document the relationship between dietary changes and improvement in PBH. This approach also allows for the early recognition and treatment of PBH. In the context of an emergency hypoglycaemic episode, it is recommended that the patient always carry fast-acting CH (dextrose or sucrose) to treat hypoglycaemia. Those treated with acarbose should avoid it. Blood glucose should be tested 15 min after consumption to confirm that the hypoglycaemic episode has resolved. If necessary, a small additional dose of CH should be administered if blood glucose remains low. Subsequently, consuming a light, balanced meal containing foods with a low-glycaemic index, rich in fibre and lean proteins, is recommended. When nutritional intervention does not yield positive results [10,12], pharmacological treatment should be attempted.

Commonly cited pharmacologic options include acarbose (often used as first line), diazoxide, calcium-channel blockers (e.g., verapamil or nifedipine), somatostatin analogues (e.g., octreotide), and SGLT2 inhibitors (e.g., empagliflozin or canagliflozin). GLP-1 receptor agonists (exenatide, liraglutide and semaglutide) have been used in PBH management in selected reports, with proposed effects on postprandial dynamics. Clinical responses appear heterogeneous and are not scientifically assumed [10,43].

The GLP-1 receptor antagonist evaluated in PBH is avexitide (exendin 9–39), which has shown efficacy in phase 2 studies [44,45]. It is not available in our country.

Endoscopic transoral outlet reduction (eTOR), using argon plasma coagulation with or without endoscopic suturing, aims to reduce the gastrojejunal anastomosis diameter after RYGB and slow gastric pouch emptying, potentially attenuating early postprandial glycemic excursions [46,47]. However, the durability of the results varies from study to study. Some studies have reported significant rates of weight regain and recurrence of symptoms, requiring re-intervention, with or without anastomotic dilation [48,49].

Current international guidance consistently frames surgery for PBH as exceptional and reserved for highly selected, medically refractory cases, given variable efficacy and the absence of consistently durable, curative outcomes across techniques [10].

A surgical approach was preferred. In revisional surgery, behavioural rehabilitation with a multidisciplinary team (MDT), including pharmacotherapy, is now recommended [10]. If this fails, more aggressive approaches, either endoscopic or surgical, may be considered. Our patient was treated accordingly, and the final decision was in favour of surgery.

Several surgical options have been considered for refractory PBH. Restrictive “add-ons” to RYGB (rings/bands) have been reported [50,51] but may carry procedure-specific risks (obstruction, erosion, bleeding, slippage). Enteral feeding via gastrostomy into the gastric remnant can attenuate PBH in selected cases by restoring duodenal nutrient passage and blunting postprandial hyperinsulinemia. However, its benefit is dependent on continued use. Symptoms usually return when oral intake is resumed.

Current recommendations do not support gastrostomy as a definitive curative strategy [10,52]. Evidence is also limited regarding its role as a physiological test before considering reversal [53]. In this case report, gastrostomy would not address weight regain.

Partial pancreatectomy is not a standard therapy for PBH and is generally reserved for rare situations in which a pancreatic source of autonomous hyperinsulinemia is diagnosed and medical therapy fails; this does not reflect typical postprandial PBH physiology [10].

Reversal of RYGB to normal anatomy has been used and may improve PBH in some patients, but recurrence of obesity-related disease and/or persistence of hypoglycemia has been reported [10,15,17,54].

Conversion to SG has also been proposed by some authors [8,55], although SG itself has been associated with PBH [13]. Comparative severity and hospitalization risk appear different across studies, but this does not establish SG as a definitive solution. From our personal experience, we have carried out a few conversions to sleeve, which have achieved clinical improvement, but not complete resolution of the clinical status.

In this case, the revisional strategy was selected to address two concurrent clinical problems: severe, disabling PBH and substantial weight regain. The intended physiological rationale was to modify nutrient delivery (pyloric pacing) and intestinal signaling (ileal stimulation) in a way expected to reduce postprandial glucose–insulin excursions and their temporal mismatch. Potential effects on enterohepatic signaling, including bile acid–FGF 19 pathways, have been proposed in mechanistic literature, although such mechanisms cannot be established from a single case.

Reports of PBH after SADI-S are limited, consisting largely of case reports or very small series; in some reports, laboratory hypoglycemia has been observed without clear clinical impact [10,36]. In a review by Palmieri et al. (2025) [56] including 1136 cases across 17 studies, no cases of hypoglycemia were reported, although heterogeneity in ascertainment and reporting limits inferences. Sánchez-Pernaute et al. (2022) [57], the innovators of the SADI-S procedure, reported the 10-year outcomes of 199 patients who underwent the procedure. The follow-up rate was 84.7% after five years and 75% after ten years; no cases of PBH were reported.

Despite the technical complexity of converting RYGB to SADI-S, the procedure was considered feasible by an experienced revisional surgery team in the current case.

### 3.7. AI Statement

Figure 1 and Figure 4 were generated with the assistance of generative AI tools.

## 4. Conclusions and Future Directions

This case suggests that, in carefully selected patients with severe post-RYGB PBH refractory to nutritional, pharmacologic and other interventions, conversion from RYGB to SADI-Sr may be a therapeutic option associated with sustained clinical improvement. In this patient, near-complete remission was observed. Only two isolated postoperative symptomatic episodes temporally associated with prolonged strenuous exercise were reported. No further episodes of PBH have been documented. However, the possibility of asymptomatic episodes must be considered, as these may occur in a significant percentage of cases [58], even if the patient is familiar with the symptoms of the condition.

Although our observations are based on a single clinical case, they reinforce the hypothesis that altering nutrient delivery and related intestinal/enterohepatic signalling can reduce the burden of PBH.

However, we cannot conclude that this represents the main aetiology, especially given the potential for other contributing factors.

As reported in the current case, a structured follow-up approach including nutritional surveillance is recommended, and an individualized risk assessment remains essential in PGB management.

Further research based on prospective studies is required to elucidate this and other potential physiopathological underlying mechanisms and the long-term efficacy and safety of all proposed therapies, including SADI-S and other ileal-based procedures.

### Patient Perspective

“Three years after the surgery, I am very satisfied because returned to my normal activity, with no more hypoglycemias and fainting fear free. Today, I notice I am in a better mood and I’m exercising four times a week, which gives me more strength for life.”

## Figures and Tables

**Table 1:** Pre-operative findings.

Exams	Key Findings	Importance
**Laboratory**	Deficiencies in ferritin, folate, vitamin B12, and vitamin D; elevated parathyroid hormone. OGTT: fasting: 93 mg/dL/120″: 50 mg/dL	Resumed intensive supplementation with iron, folate, B-complex, vitamin D.
**Barium swallow**	Normal esophagus and stomach anatomy; normal gastric emptying; no signs of reflux or hiatal hernia.	Confirmed no structural obstructions or motility issues.
**CT can**	No suspicious duodenal or pancreatic lesions or other abnormalities	No findings consistent with an insulinoma.
**Endoscopy**	No lesions or abnormalities in the esophagus, gastric stump, or intestinal loops.	Performed biopsies for further confirmation.
**Pathology**	Normal mucosa; negative for *Helicobacter pylori*; no signs of dysplasia or cancer.	Ruled out infection and malignancy.

**Table 2:** Hematological plus biochemical throughout follow-up.

Variables	Pre - op	1st PO d.	2nd PO d.	13th PO d.	30th PO d.	3rd PO m.	6th PO m.	12th PO m.	24th PO m.	36th PO m.	Reference
**Erythrocytes**	4.88	4.18	4.46	4.91	4.57	4.78	4.58	4.13	4.09	4.15	4.3–5.9 × 10^12^/L
**Hemoglobin**	13.9	12.8	13.5	14.8	13.2	14.3	13.7	13.1	13.3	13.2	13.5–17.5 g/dL
**Hematocrit**	43.1	38.5	40.7	44.2	40.2	42.5	41.8	38.6	39.5	39.6	41–53%
**INR**	0.98	----	----	1.25	----	----	----	----	0.99	1.10	0.8–1.0
**aPTT**	25.6	----	----	29.5	----	----	----	----	28.3	29.8	<28 s
**CRP**	0.27	1.85	11	2.57	1.17	0.31	0.3	----	<0.20	<0.20	<10 mg/L
**Glucose**	86	----	84	----	69	88	82	71	81	89	70–110 mg/dL
**HgbA1c**	----	----	----	----	----	4.7	4.5	4.5	4.6	4.5	≤6%
**Insulin**	27.1	----	----	----	----	----	----	----	1.3	----	2.0–25 µUI/mL
**C-peptide**	2.4	----	----	----	----	----	----	----	1.4	----	0.8–3.9 ng/mL
**Homa—IR**	6.02	----	----	----	----	----	----	----	0.25	----	1–1.9
**Homa—ß**	329.5	----	----	----	----	----	----	----	26.11	----	80–120%
**T Chol**	149	----	----	----	192	210	174	145	143	153	<200 mg/dL
**HDL**	45	----	----	----	29	39	34	38	51	51	30–70 mg/dL
**T Chol/HDL**	3.3	----	----	----	6.6	5.4	5.1	3.8	2.8	----	<4.5 mg/dL
**Triglycerides**	83	----	----	----	123	136	105	66	61	65	35–160 mg/dL
**AST**	25	196	64	----	19	17	16	20	18	20	15–40 U/L
**ALT**	37	269	109	----	19	20	16	20	17	22	10–40 U/L
**Alkaline phosphatase**	----	----	----	119	94	71	72	72	74	63	30–100 U/L
**GGT**	----	----	----	82	39	24	26	20	18	17	8–78 U/L
**LDH**	----	362	113	----	----	----	----	----	----	----	45–90 U/L
**CPK**	----	543	63	**----**	----	----	----	----	----	----	55–170 U/L
**Total bilirubin**	----	0.39	0.39	**----**	----	----	----	----	----	0.72	0.1–1 mg/dL
**Conjugated bilirubin**	----	0.2	0.2	**----**	----	----	----	----	----	----	<0.3 mg/dL
**Urea**	----	44	----	**----**	----	----	----	----	----	46	15–45 mg/dL
**Creatinine**	----	0.99	0.79	**----**	1.16	----	----	0,99	----	0.98	0.6–1.2 mg/dL
**Sodium (Na)**	137	137	137	**----**	143	141	144	142	142	142	135–146 mEq/L
**Potassium (K)**	4.5	4.4	4.2	**----**	4.2	3.9	4.4	4.3	4.2	4.3	3.5–5 mEq/L
**Chlorine (Cl)**	103	101	102	**----**	109	109	106	106	106	107	95–105 mEq/L

**Table 3:** Nutritional deficiencies throughout follow-up.

Variables	Preop	3rd PO m.	6th PO m.	12th PO m.	24th PO m.	36th PO m.
**Total Proteins**	6.4	6.5	6.8	6.4	6.5	6.6
**Albumin**	3.9	4.2	4.3	4.1	4.4	4.5
**Iron**	94	99	93	115	138	130
**Ferritin**	21	20	34	47.8	87.2	72.6
**Vitamin B9**	2.7	-----	-----	5.54	7.34	7.46
**Vitamin B12**	272	495	750	962	863	762
**Vitamin D**	17	-----	-----	19.5	13	13.7
**Parathormone**	93	-----	94	68.6	38.2	45.1
**Calcium**	8.9	9.0	9.2	9.3	9.4	-----
**Phosphorus**	3.2	2.7	2.8	3.2	3.8	-----
**Magnesium**	2.2	-----	-----	2.1	1.9	-----

## Data Availability

The data that supports the findings of this study are available on request from the corresponding author.
